# Thin Functional Polymer Films by Electropolymerization

**DOI:** 10.3390/nano9081125

**Published:** 2019-08-04

**Authors:** Alex Palma-Cando, Ibeth Rendón-Enríquez, Michael Tausch, Ullrich Scherf

**Affiliations:** 1 School of Chemical Sciences and Engineering, Universidad Yachay Tech, EC100115 Urcuqui, Ecuador; 2 Macromolecular Chemistry Group, Bergische Universität Wuppertal, Gaußstraße 20, D-42119 Wuppertal, Germany; 3 Department of Chemistry and Chemical Education, Bergische Universität Wuppertal, Gaußstraße 20, D-42119 Wuppertal, Germany

**Keywords:** electropolymerization, thin films, electrochromic devices, microporosity, polymer networks, nitrobenzene detection

## Abstract

Intrinsically conducting polymers (ICPs) have been widely utilized in organic electronics, actuators, electrochromic devices, and sensors. Many potential applications demand the formation of thin polymer films, which can be generated by electrochemical polymerization. Electrochemical methods are quite powerful and versatile and can be utilized for investigation of ICPs, both for educational purposes and materials chemistry research. In this study, we show that potentiodynamic and potentiostatic techniques can be utilized for generating and characterizing thin polymer films under the context of educational chemistry research and state-of-the-art polymer research. First, two well-known bifunctional monomers (with only two linking sites)—aniline and bithiophene—and their respective ICPs—polyaniline (PANI) and polybithiophene (PBTh)—were electrochemically generated and characterized. Tests with simple electrochromic devices based on PANI and PBTh were carried out at different doping levels, where changes in the UV-VIS absorption spectra and color were ascribed to changes in the polymer structures. These experiments may attract students’ interest in the electrochemical polymerization of ICPs as doping/dedoping processes can be easily understood from observable color changes to the naked eye, as shown for the two polymers. Second, two new carbazole-based multifunctional monomers (with three or more linking sites)—tris(4-(carbazol-9-yl)phenyl)silanol (TPTCzSiOH) and tris(3,5-di(carbazol-9-yl)phenyl)silanol (TPHxCzSiOH)—were synthesized to produce thin films of cross-linked polymer networks by electropolymerization. These thin polymer films were characterized by electrochemical quartz crystal microbalance (EQCM) experiments and nitrogen sorption, and the results showed a microporous nature with high specific surface areas up to 930 m^2^g^−1^. PTPHxCzSiOH-modified glassy carbon electrodes showed an enhanced electrochemical response to nitrobenzene as prototypical nitroaromatic compound compared to unmodified glassy carbon electrodes.

## 1. Introduction

Since the dawn of the intrinsically conducting polymers (ICPs) in the late 1970s [1,2], electrochemistry has been a powerful tool, not only for synthesis of polymer films but also for in situ characterization of (semi)conducting materials [3,4]. Electrochemical generation of ICPs is based mainly on the oxidative condensation of different aromatic heterocycles and derivatives through the formation of radical cations [5]. Pyrrole, aniline, thiophene, and carbazole are among the simplest molecules that can be electrochemically polymerized due to their inherent low oxidation potential [6]. Corresponding electrosynthesized ICPs have shown potential applications in electrocatalysis, batteries, capacitors, sensors, biosensors, organic light-emitting diodes, organic solar cells, and electrochromic devices [7]. The principle of the last application, i.e., electrochromism, could be in fact an interesting and innovative approach to be used in undergraduate and graduate classes [8,9]. Explanation of the electrochemical doping/dedoping processes of ICPs can be easily managed by applying different potentials to polymer films, resulting in color changes of the deposits via a suited modification of their chemical and electronic structures [10,11,12,13,14]. We have previously reported electrochemical polymerization of three different monomers—4,7-dithienyl-2,1,3-benzothiadiazole, 3,4-ethilendioxythiophene, and pyrrole—using low-cost potentiostats [15,16]. Electrochromism of the synthesized polymer films could be easily demonstrated by fabrication of electrochromic windows. These optoelectronic devices showed a clear change in UV-VIS spectra and color when positive or negative potentials were applied. The presented methodology is well suited for the development of experiments in undergraduate and graduate education for demonstrating and explaining electrochromicity.

Electrochemical polymerization of bifunctional monomers (with only two linkable sites) results mainly in the formation of linear, 1D conducting polymer films that have unique advantages over inorganic semiconductors, such as one-step synthesis, thin film deposition, light weight, and low cost [17,18]. However, the formed conjugated polymer chains are usually twisted and entangled, leading to a disordered solid-state structure that might result in lower performance in applications where high order is required (e.g., organic electronics). On this ground, over the past years, electrochemical generation of polymer films has been intensively studied, starting from multifunctional monomers (with three or more linkable sites) [19,20,21,22,23]. This methodology usually leads to the formation of films with a 3D network structure that not only maintains all advantages of 1D polymer films but also shows a more robust electronic structure, often accompanied by the occurrence of inherent microporosity with high surface area values [24,25,26]. Our group has previously synthesized different multifunctional monomers (with up to eight linkable sites) with different cores and linkers that allow the establishment of reliable structure–property relationships between the number of functionalities and the specific surface area of the corresponding electrochemically generated microporous polymer networks (MPNs) [27,28]. Semiconductivity and high surface areas of >2000 m^2^g^−1^, in some cases, for these microporous, electron-rich polymer films allowed us to explore their potential applications as electrochemical or fluorescence sensors for nitroaromatic explosives that showed an improved sensitivity by increasing the number of available active sites for molecular interaction [29,30]. In the context of the results of this report, Zhang et al. recently reviewed the electrochemical generation of polymer films using bifunctional or multifunctional monomers and their potential applications [31]. Our study tries to link educational chemistry and materials chemistry, both dealing with the electropolymerization of bi- or higher-functional monomers into functional polymer films.

**Scheme 1:**
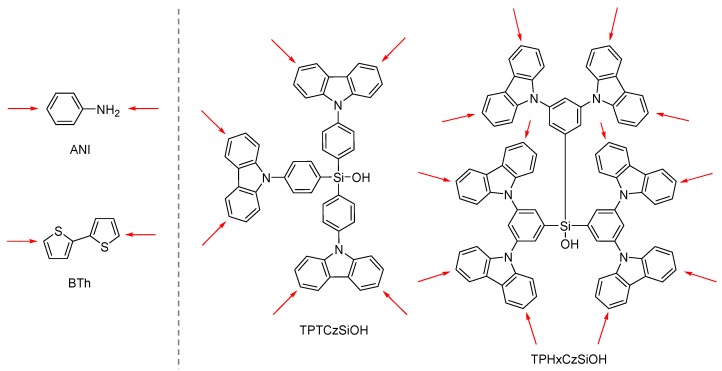
Chemical structures of the electropolymerized bifunctional (**left**) and multifunctional (**right**) monomers. The arrows show the available linking sites on each molecule.

In this study, we present the electrochemical polymerization of two well-known bifunctional monomers—aniline and bithiophene—in the context of educational chemistry research (see Scheme 1). Electrochemical and spectrophotometric characterization was carried out for the prepared polymer films. Construction of electrochromic windows based on polyaniline (PANI) and polybithiophene (PBTh) was carried out using a very simple setup. At different doping levels, changes in their UV-VIS absorption spectra and color (green-blue-yellow for PANI and pink-blue for PBTh) were observed, which were ascribed to changes in the polymer structures. On the other hand, two new carbazole-based multifunctional monomers—tris(4-(carbazol-9-yl)phenyl)silanol (TPTCzSiOH) and tris(3,5-di(carbazol-9-yl)phenyl)silanol (TPHxCzSiOH) (see Scheme 1)—were electropolymerized into cross-linked, microporous, functional polymer films as an example for the current topic of materials chemistry. Moreover, the electrochemical formation of microporous thin films from multifunctional carbazole monomers was followed up with the electrochemical quartz crystal microbalance (EQCM) method. High specific surface areas (S_BET_) for both films were determined, with PTPHxCzSiOH films showing a S_BET_ of 930 m^2^g^−1^. PTPHxCzSiOH-modified glassy carbon (GC) electrodes showed improved electrochemical detection ability for nitrobenzene as prototypical nitroaromatic analyte compared to bare GC electrode response. Thus, the study merges electropolymerization research in two areas by reporting original results (i) on the adaptation of electrochemical ICP synthesis setup and characterization methods for educational chemistry needs and for a presentation of structure–properties relationship in classroom experiments and (ii) on the electrochemical generation of microporous polymer films, starting from two novel multifunctional monomers.

## 2. Materials and Methods 

### 2.1. Construction of the Electrochromic Devices Based on 1D Polymer Films for Educational Chemistry Purposes 

Electrodeposition of PANI was performed in an aqueous 0.4 M aniline solution with 0.5 M sulfuric acid, while electrodeposition of PBTh was made in a 10 mM 2,2′-bithiophene solution in acetonitrile (MeCN) with 0.1 M of tetrabutylammonium perchlorate (TBAP). Fluorine-doped tin oxide (FTO) electrodes (3.5 cm × 3.5 cm) were used as working electrodes (WE), and stainless-steel plates (3.5 cm × 3.5 cm) were used as counter electrodes (CE). For potentiostatic electrodeposition of PANI and PBTh films, constant potentials of 0.7 V vs. Ag/AgCl and 0.85 V vs. Ag/AgNO_3_ were applied for 5 min, respectively. The electrolyte solution was prepared in a small brown glass vial using 1 mL of propylene carbonate (PC), 1.3 g of poly(ethylene glycol) diacrylate (PDA), 5.5 mg of 2,2-dimethoxy-2-phenylacetopheneone (DMAP), and 0.2 g of lithium trifluoromethanesulfonate (LITRIF). Subsequently, the mixture was treated for 15 min in an ultrasound bath. Two drops of this solution were added into the polymer-modified FTO electrode. A second clean FTO was placed on the top with a separation distance between the electrodes of ca. 3 mm. Photopolymerization of PDA was done under illumination with 365 nm light for 15 min to generate the gel electrolyte layer. Doping and dedoping potentials were applied between the electrodes to evaluate electrochromism of these devices. 

The material safety data sheets should be consulted if appropriate. A short list of hazards for all these chemicals can be found in the Supplementary Material. 

### 2.2. Electrochemical Synthesis and Response to Nitrobenzene (NB) of Microporous 3D Polymer Films as Current Topic in Materials Chemistry

Ten milliliters of 0.1 mM solutions of TPTCzSiOH and TPHxCzSiOH were placed in a three-electrode cell at 25 °C. Glassy carbon working electrodes were connected to platinum wire counter electrodes. Ag°/AgNO3 was used as nonaqueous reference electrode (RE). Potentiostatic polymerization of TPTCzSiOH and TPHxCzSiOH was attained under inert atmosphere by applying a constant polymerization potential of 1.1 V until an oxidative charge of 0.20 mC was accumulated. After electrochemical dedoping and washing of the deposits with MeCN and methylene chloride, the polymer-modified GC electrodes were placed in 10 mL of an aqueous solution containing 0.2 M potassium chloride and 0.1 M phosphate buffer under inert atmosphere at 25 °C. An aqueous reference electrode (Ag°/AgCl) was used for these tests. Equilibration was achieved by stirring the mixtures for 5 min followed by applying a prepotential of 0 V for 30 s. Linear scan voltammograms (LSVs) were registered in the potential range of 0 V to −1 V with a scan rate of 10 mV s^−1^. For detection of nitroaromatic compounds, aliquots from a 100 M nitrobenzene (NB) stock solution in MeCN were added to the buffered solutions to reach the intended NB concentration (0.5 μM). 

For additional and detailed information, please see the Supplementary Material. 

## 3. Results and Discussion

### 3.1. Electrochemical Polymerization of Bifunctional Monomers for a Simple Demonstration of Electrochromic Effects

**Figure 1:**
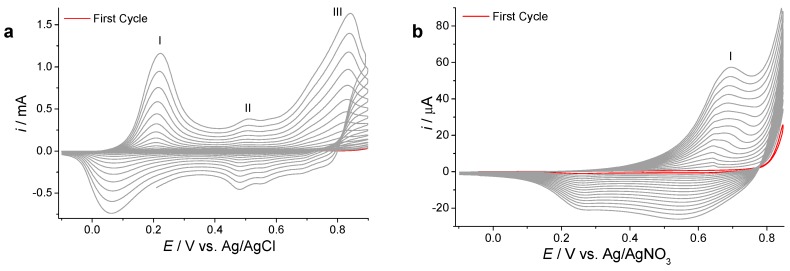
(**a**) Cyclic voltammograms (CVs) for electropolymerization of 0.4 M aniline in an aqueous 0.5 M sulfuric acid solution at platinum disc electrodes. (**b**) Cyclic voltammograms for electropolymerization of 2 mM bithiophene and 0.1 M tetrabutylammonium perchlorate solutions in MeCN at platinum disc electrodes. The voltammograms were recorder from −0.1 to 0.9 V (polyaniline (PANI)), or 0.85 V (polybithiophene (PBTh)) with a sweep rate of 0.1 V s^−1^.

The electrochemical polymerization of aniline proceeds via a radical propagation mechanism. The initial step is the oxidation of two molecules of aniline to radical cations. The second step involvs a radical coupling between the N-radical cation and *para*-carbon radical to form a dimer, which further undergoes proton elimination. Chain propagation involves oxidation of dimer to a dimer N-radical cation and coupling with *para*-carbon radical to form a trimer after deprotonation. This cascade process leads to deposition of PANI on the surface of an electrode driven by the reduced solubility of the coupling products (see Supplementary Scheme S1). Unlike in other conductive polymers, the heteroatom is directly introduced as a linker atom into the main chain [32]. Using cyclic voltammetry, electrodeposition of PANI was evaluated on the surface of a platinum disc electrode (see Figure 1a). Polymerization under potentiodynamic conditions provided information about the growth of the film. Oxidation of the monomer began at 0.85 V (red line in Figure 1a), while oxidation of the polymer showed three peaks. Peak I (ca. 0.2 V) corresponded to the first step of oxidation of the species HN–R–NH (R: C_6_H_4_) in the main chain to a cation radical or polaron system HN–R–NH^+^. The cation radical was then oxidized, almost at the same potential, to the dication or bipolaron HN^+^ = R = NH^+^. The dication could then be oxidized at a potential that corresponded to the third peak of ca. 0.85 V [33]. The middle peak in the voltammogram was related to the presence of an intermediate during the electropolymerization of aniline and/or to the formation of cross-linked polyaniline chains [34]. The increase in the current with every next cycle of multisweep cyclic voltammograms (CVs) showed an increase in the polymer thickness as well as in the number of redox-active sites. 

The electropolymerization of bithiophene also proceeds via a radical propagation mechanism. The polymerization starts in solution and takes place from a dimer oxidation to a tetramer and so on until deposition of elongated polymer chains (see Supplementary Scheme S2) [5]. Therefore, electrodeposition of polybithiophene (PBTh) films could be accomplished by applying oxidative potentials to a diluted solution of 2,2′-bithiophene (see Figure 1b) [35]. Oxidation of the monomer began at 0.81 V (red line in Figure 1b). Peak I, around 0.68 V, was assigned to p-doping of the as-prepared polymer chains, which appeared from the second sweep onwards. The two reduction peaks (0.65 V and 0.25 V) were assigned to two stable states of partially discharged polymer chains [36]. The generated polymer (polybithiophene) was of high stability as the charging and discharging processes were fully reversible. In other words, the ratio between the negative charge (Q− discharging process) and the positive charge (Q+ charging process) was around one in each cycle [37,38,39]. The conducted experiments showed good stability for both polymer films under the applied conditions (see Supplementary Figure S1).

**Scheme 2:**
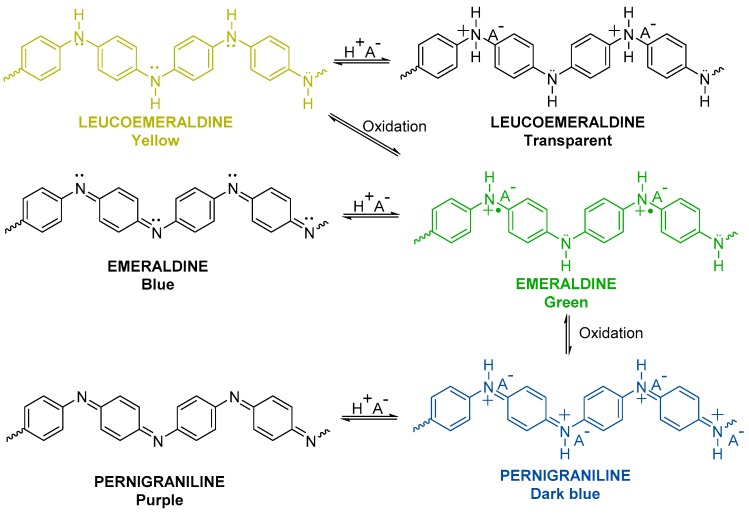
Protonation/deprotonation and redox reactions between the discussed redox states of polyaniline.

Both polymers show a distinct electrochromism behavior, i.e., the film changed color when different redox potentials were applied. In the case of PANI, it is possible to differentiate three chemical and electronic structures—leucoemeraldine, emeraldine, and pernigraniline—all showing different colors and electronic properties (see Scheme 2) [32,40,41].

**Figure 2:**
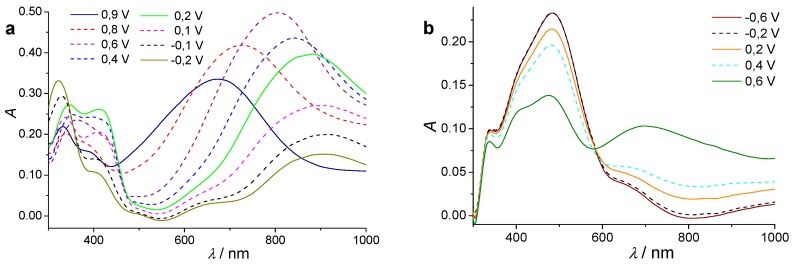
UV-VIS absorption spectra of (**a**) PANI and (**b**) PBTh deposited on fluorine-doped tin oxide (FTO) at different doping levels. Polymer films were placed in a 0.1 M KCl aqueous solution (for PANI-modified electrode) and 0.1 M tetrabutylammonium perchlorate solutions in MeCN (for PBTh-modified electrode) followed by application of different potentials for 10 s.

In our experiments, UV-VIS absorption spectra were measured for both polymers after different potentials were applied in 0.1 M KCl aqueous solution (for PANI-modified electrodes) and 0.1 M of tetrabutylammonium perchlorate in MeCN solution (for PBTh-modified electrodes) (see Figure 2). All forms of PANI showed two clear absorption bands; the first one was observed with a maximum at about 350 nm and a shoulder around 400 nm. According to the literature, this band was related to a π − π* transition [41]. The second band arose from 500 to 1000 nm, which was related to the formation of polarons or radical cations (polaron band). The applications of low potentials, ca. −0.2 V, led to the formation of the leucoemeraldine form of PANI. Starting from −0.2 V, as the applied potential increased up to 0.2 V, the absorption maximum at around 350 nm also increased, the shoulder at ca. 400 nm decreased, and there was also a bathochromic shift for both peaks. Additionally, the polaron band absorption increased with the rise of the applied potential to PANI-modified electrode adopting the emeraldine structure. The peak at 350 nm continuously decreased with increasing applied potential up to 0.6 V. The polaron band also showed a hypochromic shift. By increasing the applied potential up to 0.9 V, the polaron band further shifted hypochromically and started to decrease with the formation of the pernigraniline structure [40]. A PBTh film showed an absorption maximum around 500 nm with two shoulders at ca. 350 and 400 nm. This band was related to a π − π* transition in the polymer chain, which decreased as the applied potential increased [42]. There was a second broad band with a maximum at about 700 nm ascribed to an excitation of polarons or radical cations (polaron band). The intensity of the polaron band increased as the applied potential was increased. The polaronic state appeared at applied potentials between 0.2 and 0.4 V. The application of a potential of 0.6 V led to the formation of bipolaronic structures [42]. PBTh film could be dedoped into the neutral state when a negative potential in the range of −0.6 to −0.2 V was applied. 

**Figure 3:**
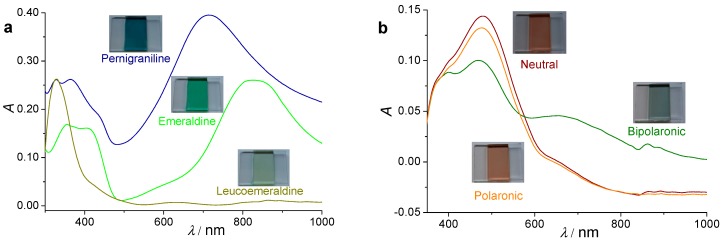
Absorption spectra and photographic images of electrochromic devices based on (**a**) PANI and (**b**) PBTh at different doped levels.

Electrochromism of these thin polymer films can be exploited for teaching purposes by building low-cost electrochromic devices made of electropolymerized active layers. These optoelectronic devices can easily demonstrate correlation between chemical structure and doping level of polymers at a certain potential by visual color changes. Herein, a solution of DMAP (0.02 M) and LITRIF (0.10 M) in PC/PDA (1:1) as electrolyte precursor was prepared [43], and two drops of this solution were placed onto a FTO electrode with an electrodeposited thin polymer film. A second FTO plate was used as counter electrode. The photopolymerization of PDA under illumination with 365 nm light kept the two electrodes together by formation of a gel electrolyte. Different potentials were applied between the electrodes, leading to changes in the color of the devices and their absorption spectra (see Figure 3). The potential was applied using a low-cost potentiostat without a reference electrode [15,16]. After the construction of the device, PANI films remained partially oxidized on its polaronic state, which corresponded to the emeraldine form (see Figure 3a). The polymer can suffer further oxidation to a pernigraniline form on its bipolaronic state and take a blue color. If a reductive potential is applied, the polymer reaches its neutral state or leucoemeraldine form with a yellow color. PBTh films remained partially oxidized on its polaronic state after the construction of the device, taking a light pink color (see Figure 3b). The polymer can suffer further oxidation to its bipolaronic state with a green color. If a reductive potential is applied, PBTh reaches its neutral form and changes color to dark pink. 

### 3.2. Electrochemical Polymerization of Multifunctional Monomers as a Current Topic for the Electrodeposition of Functional Films

The silicon-centered tri- or hexafunctional monomers were prepared according to previously reported procedures. Synthetic details and structural characterization of both monomers are presented in the Supplementary Material. Partial coupling (trisubstitution) of SiCl_4_ with 9-(4-bromophenyl)carbazole or 5-bromo-1,3-di(carbazol-9-yl)benzene was realized with ca. 30% yield to give TPTCzSiOH or TPHxCzSiOH monomers, respectively.

**Figure 4:**
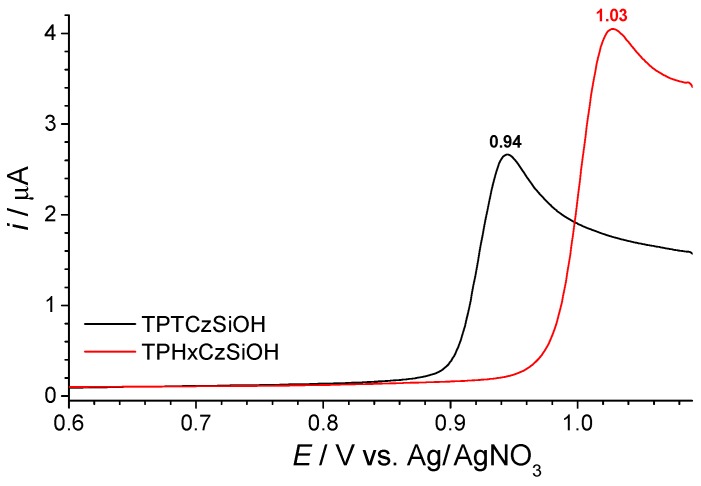
First anodic sweep voltammograms for 0.1 mM of tris(4-(carbazol-9-yl)phenyl)silanol (TPTCzSiOH) (black line) or tris(3,5-di(carbazol-9-yl)phenyl)silanol (TPHxCzSiOH) (red line) and 0.1 M tetrabutylammonium perchlorate solutions in MeCN/methylene chloride (1:4) at platinum disc electrodes. The voltammograms were recorded from 0 to 1.1 V with a sweep rate of 0.10 V s^−1^.

MPN film generation was achieved by electrochemical oxidative coupling of both carbazole derivatives. For these experiments, 0.1 mM solutions of the monomers were prepared in MeCN/methylene chloride (1:4). Tetrabutylammonium perchlorate (TBAP) was added to the solutions as supporting electrolyte with a concentration of 0.1 M. A conventional three-electrode cell was set up with platinum disc as working electrode, platinum wire as counter electrode, and Ag/Ag^+^ as reference electrode. Figure 4 shows the first anodic voltammograms up to 1.1 V at a sweep rate of 0.10 V s^−1^ for electropolymerization of TPTCzSiOH (black line) and TPHxCzSiOH (red line) on platinum disc electrodes. First oxidation peaks were attributed to a cascade of oxidation of carbazoles into radical cations followed by coupling and deprotonation of the intermediates into 3,3′-dicarbazole dimers [44,45]. TPHxCzSiOH oxidation current peak was higher than TPTCzSiOH oxidation current peak in line with double the amount of electroactive groups. Higher oxidation potential was needed for the hexacarbazolyl monomer compared to the tricarbazolyl monomer, which could be related to the sterically demanding di(carbazol-9-yl)-benzene linker, resulting in a higher carbazolyl−phenyl distortion. Twenty successive CVs were recorded from −1.0 to 1.1 V at a sweep rate of 0.10 V s^−1^ for polymerization of TPTCzSiOH and TPHxCzSiOH (see Supplementary Figure S2). Successive sweeping prompted a gradually increased current, indicating a cumulative polymer deposit on the electrode. In both cases, two reversible peaks were clearly shown in potentials from 0.4 to 1.1 V. These waves could be related to the charging/discharging of the MPN films by formation of radical cations (polarons) or dications (bipolarons) [46]. MPN-modified platinum disc electrodes were studied in monomer-free solutions by recording CVs at different sweep rates (see Supplementary Figure S3). A linear relationship was shown between anodic/cathodic peak current and sweep rate, which is characteristic of well-adhered thin deposits with absence of diffusional limitations [47].

**Figure 5:**
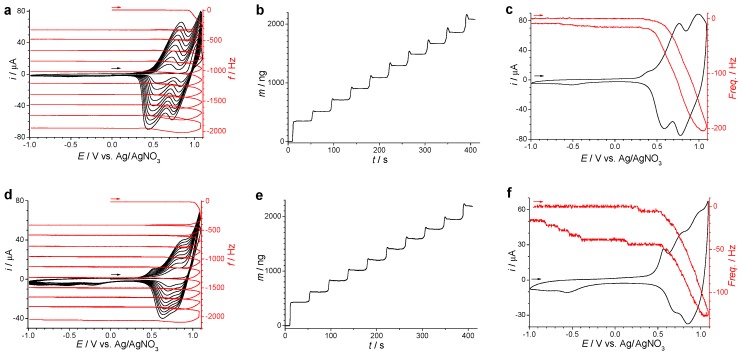
Ten successive CVs (black lines) and corresponding quartz microbalance frequency response (red lines) in the electropolymerization at platinum/quartz working electrodes of 0.1 mM solutions of (**a**) TPTCzSiOH and (**d**) TPHxCzSiOH and 0.1 M tetrabutylammonium perchlorate in MeCN/methylene chloride (1:4). Mass accumulation during electropolymerization of (**b**) TPTCzSiOH and (**e**) TPHxCzSiOH, calculated by applying the Sauerbrey equation. CVs (black lines) and corresponding quartz microbalance frequency response (red lines) for thin films of (**c**) PTPTCzSiOH and (**f**) PTPHxCzSiOH on platinum/quartz electrodes in 0.1 M tetrabutylammonium perchlorate in MeCN solutions. All CVs were registered from −1.0 to 1.1 V with a sweep rate of 0.10 V s^−1^.

EQCM gives additional information regarding gain and loss of material on top of an electrode by determination of frequency changes [48]. The frequency variations (f) of a quartz crystal resonator is associated with the mass change by the Sauerbrey relation [49]. Ten cyclic voltammograms were recorded for TPTCzSiOH and TPHxCzSiOH electropolymerization at platinum/quartz electrodes under identical settings as previously outlined (see Figure 5a,d). During the first anodic sweep, onset frequencies decrease clearly corresponded to respective oxidation onset potentials of TPTCzSiOH (ca. 0.9 V) and TPHxCzSiOH (ca. 1.0 V), where polymer deposition started. Progressive polymer film formation led to continuously decrease frequency values. This phenomenon can be better appreciated in a mass vs. time plot (see Figure 5b,e). A constantly increasing mass was determined for a possible layer-by-layer film growth. Peaks observed at the beginning of each mass step were related to the reversible counteranion uptake and release in the charging/discharging process of the electroactive polymer films. This reversible process was observed well by recording cyclic voltammograms coupled to QCM for polymer deposits in monomer-free solutions (0.1 M tetrabutylammonium perchlorate in MeCN) (see Figure 5c,f). Polymer oxidation (p-doping) process was accompanied with insertion of perchlorate counteranions into the deposited matrix, resulting in a progressive frequency decrease (e.g., mass increase). Subsequent polymer reduction (dedoping) corresponded to a release of perchlorate counter anions from the polymer matrix into the solution, resulting in frequency increase (e.g., mass decrease). Frequency hysteresis and slight mass growth at the switching potential of the cycle might be connected to a restricted counterion mobility and to a partial release of trapped perchlorate counterions, respectively [50].

**Figure 6:**
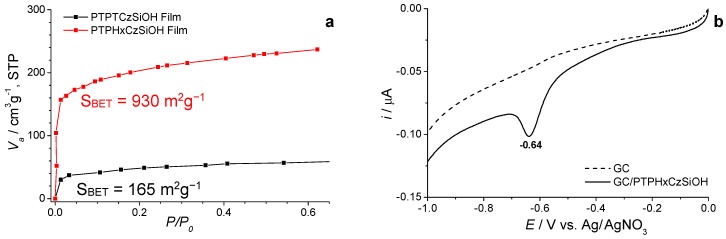
(**a**) Adsorption isotherms of electrochemically produced polymer films using krypton gas. Specific surface areas were obtained by applying the Brunauer−Emmett−Teller (BET) equation to the krypton gas adsorption isotherms (mean value of three measurements). (**b**) Linear scan voltammograms for the detection of 0.5 μM nitrobenzene at bare (dashed line) and PTPHxCzSiOH-modified (solid line) glassy carbon electrodes in aqueous, buffered solutions. These voltammograms were recorded with a sweep rate of 0.01 V s^−1^.

Morphology studies of thin MPN films were carried out by tapping-mode atomic force microscopy (AFM) analysis (see Supplementary Figure S4 and Table S1). These films showed average surface roughness (R_q_)/thickness of 8.0 nm/45 nm for PTPTCzSiOH and 6.3 nm/50 nm for PTPHxCzSiOH, respectively. Specific surface areas of the porous films were calculated from the polymer’s krypton adsorption isotherms in the relative pressure range of 0–0.6 at 77 K (see Figure 6a). Thick, free-standing films were obtained on indium tin oxide (ITO) electrodes by potentiostatic electropolymerization applying 1.0 V (in 0.5 mM TPTCzSiOH solution) and 1.1 V (in 0.5 mM TPHxCzSiOH solution) for 20 min. High gas adsorption at low relative pressure (<0.1) indicates microporosity of the polymer films [51]. Specific surface areas (S_BET_) of 165 m^2^g^−1^ for PTPTCzSiOH films and 930 m^2^g^−1^ for PTPHxCzSiOH films were computed using the Brunauer−Emmett−Teller (BET) equation. Higher surface areas are usually expected for higher monomer functionality due to an increased cross-linking density and rigidity, resulting in an increased permanent microporosity [25]. For comparison purposes, chemical oxidative polymerization of both monomers was carried out with iron(III) chloride, resulting in the formation of powdery polymers in good yields (>85%). These materials showed good thermal (see Supplementary Figure S5) and chemical resistance and strong blue photoluminescence (see Supplementary Figure S6). Nitrogen sorption isotherms at 77 K were recorded for these chemically made bulk polymers (see Supplementary Figure S7), showing similar trends in the adsorption isotherms for films and bulk materials. Slightly reduced specific surface areas were usually found for the electrochemically made polymer films (165 m^2^g^−1^ for PTPTCzSiOH films and 930 m^2^g^−1^ for PTPHxCzSiOH films) compared to chemically made powdery materials (405 m^2^g^−1^ for PTPTCzSiOH powder and 1285 m^2^g^−1^ for PTPHxCzSiOH powder). This phenomenon may be ascribed to a reduced cross-linking density during electropolymerization compared to the FeCl_3_-mediated condensation. Electrochemical oxidative coupling of carbazoles leads mainly to the formation of 3,3′-dimers, while chemical oxidative coupling might also form additional 7,7′-dicarbazole linkages [52]. 

Finally, the electrochemical response of nitrobenzene (NB) as prototypical nitroaromatic analyte was demonstrated on GC electrodes coated with microporous PTPHxCzSiOH deposits. Microporous polymer films were obtained by potentiostatic oxidative polymerization of 0.1 mM TPHxCzSiOH in MeCN/methylene chloride (1:4) solutions. The films were subsequently washed and transferred into an aqueous environment. Figure 6b shows linear scan voltammograms (LSVs) for the reduction of 0.5 μM NB on bare glassy carbon and PTPHxCzSiOH-modified electrodes. These LSVs were registered in aqueous, buffered solutions (pH 7.4) in a potential window from 0 to −1.0 V with a sweep rate of 0.01 V s^−1^. A single wave occurred at a potential of −0.64 V, which corresponded to the reduction of nitro into hydroxylamine groups as a four-electron reduction of nitrobenzene [53]. In contrast, no detectable reduction peak was observed for the reduction of 0.5 μM NB on bare GC electrodes, thus demonstrating the increased detection of the porous film-modified electrodes. This observation can be explained by increased interfacial interactions between the electron-rich microporous polymers and the electron-poor nitroaromatic analytes [54].

## 4. Conclusions

Two different application scenarios were outlined for electrochemically deposited thin polymer films. Electrochemically generated polyaniline or polybithiophene films (made from bifunctional monomers) led to the formation of 1D polymer chains. Films of these polymers showed clear color changes at different doping levels. Electrochromicity experiments with these films can support teaching classes related to the electroactivity of organic semiconductors. TPTCzSiOH and TPHxCzSiOH (multifunctional monomers) were utilized for the electrodeposition of microporous polymer films as an example of current polymer electrochemistry research. High intrinsic surface areas of these electron-rich polymer films motivated us to successfully use them for the detection of electron-poor nitroaromatic analytes in micromolar levels.

## Supplementary Materials

The following is available online at https://www.mdpi.com/2079-4991/9/8/1125/s1, Synthetic details, electrochemical, optical, thermal, morphological, and porosity characterization of the polymer materials. Scheme S1: Electrochemical polymerization steps of aniline condensation during deposition of PANI films, Scheme S2: Electrochemical polymerization steps of bithiophene during deposition of PBTh films, Figure S1: Cathodic to anodic charging/discharging ratios for 30 sweeps during deposition of PANI and PBTh onto platinum disc electrodes, Figure S2: Polymerization CVs of TPTCzSiOH and TPHxCzSiOH monomers at platinum disc electrodes, Figure S3: CVs in monomer-free solution and linear dependence of peaks current and sweep rate for PTPTCzSiOH and PTPHxCzSiOH deposits, Figure S4: AFM images of thin, microporous polymer films on ITO, Table S1: Average roughness/thicknesses values of electrogenerated thin porous polymer films on ITO, Figure S5: TGA plots of the corresponding chemically synthesized, bulk polymer networks, Figure S6: UV-VIS and photoluminescence spectra of the bulk polymer networks, Figure S7: Nitrogen gas sorption isotherms and specific surface areas of bulk polymer networks.
