# Supplementary material for: Thin Functional Polymer Films by Electropolymerization
Source: Nanomaterials (Basel). 2019 Aug 4;9(8):1125. doi: 10.3390/nano9081125 (PMC6723103; doi:10.3390/nano9081125)
Supplement: Supplementary file 1 [file nanomaterials-09-01125-s001.pdf]

Supplementary Material

Thin Functional Polymer Films by Electropolymerization

Alex Palma-Cando [1,2,*], Ibeth Rendón-Enríquez [3], Michael Tausch [3,*] and Ullrich Scherf [2,*]

[1] School of Chemical Sciences and Engineering, Universidad Yachay Tech, EC100115 Urcuqui, Ecuador
[2] Macromolecular Chemistry Group, Bergische Universität Wuppertal, Gaußstraße 20, D-42119 Wuppertal, Germany
[3] Department of Chemistry and Chemical Education, Bergische Universität Wuppertal, Gaußstraße 20, D-42119 Wuppertal, Germany
* Correspondence: apalma@yachaytech.edu.ec (A.P.-C.); mtausch@uni-wuppertal.de (M.T.); scherf@uni-wuppertal.de (U.S.)

Materials and Methods

Electropolymerization in the Context of Educational Chemistry

Electrochemical Generation of 1D Polymer Films and Subsequent Characterization

Electrodeposition of polyaniline (PANI) was performed in an aqueous 0.4 M aniline solution (≥99%, Merck) with 0.5 M sulfuric acid while electrodeposition of polybithiophene (PBTh) was made in a 10 mM 2,2'-bithiophene (98%, ABCR) solution in acetonitrile with 0.1 M of tetrabutylammonium perchlorate (TBAP, for electrochemistry, FLUKA). Experiments were conducted using a three-electrode cell, with platinum disc electrode as working electrode (WE), a platinum wire as counter electrode (CE) and Ag/AgCl as reference electrode (RE, $KCl_{(sat)}$ 0,20 V vs SHE) for PANI deposition and $Ag/AgNO_3$ electrode (0.1 M $AgNO_3$, 0.60 V vs SHE) for PBTh generation. The electrodeposition was accomplished with 15 potentiodynamic cycles with a scan rate of 0.1 V s^{-1}. After that, cyclic voltammograms were performed for polymer modified electrodes in monomer-free solution during 30 cycles with the same scan rate. For every cycle, the polymer charge was determined during the charging and discharging redox processes.

Construction of electrochromic devices based on 1D-polymer films

Electrochemical generation of thin polymer films was carried out using monomer solutions as previously described. FTO electrodes (3.5 cm x 3.5 cm) were used as WE and stainless-steel plate (3.5 cm x 3.5 cm) as CE. For potentiostatic electrodeposition of PANI and PBTh films, constant potentials of 0.7 V vs. Ag/AgCl, or 0.85 V vs. Ag/Ag NO_3, respectively, were applied for 5 minutes. Electrochromic behaviour of each polymer film was recorded by UV-Vis spectrophotometry at different potentials. Doping and dedoping experiments were made in an aqueous 0.1 M hydrochloric acid solution for PANI, or in an acetonitrile 0.1 M TBAP solution for PBTh.

To build electrochromic windows, the following substances were used: propylene carbonate (PC, Alfa Aesar), poly(ethylene glycol) diacrylate (PDA, Molecular weight M_n: 700 g/mol, Sigma Aldrich), 2,2-dimethoxy-2-phenylacetopheneone (DMAP, TCI), and lithium trifluoromethanesulfonate (LITRIF, TCI).

Electrolyte solution was prepared in a small brown glass vial using 1 mL of PC, 1.3 g of PDA, 5.5 mg of DMAP, and 0.2 g of LITRIF. Subsequently, the mixture was treated for 15 minutes in an ultrasound bath.[11] Two drops of this solution were deposited onto the polymer modified FTO-electrode. A second clean FTO was placed on top with a separation distance between the electrodes of ca. 3 mm. Photopolymerization of PDA was accomplished by illumination with 365 nm light for 15 minutes in order to generate the gel electrolyte layer. Doping and dedoping potentials were applied between the electrodes to evaluate the electrochromic properties of these devices.

Hazards: **Aniline** is toxic if inhaled, swallowed and in contact with the skin. It causes damage to blood system through prolonged or repeated exposure as well as serious eye damage, allergic and skin reaction. It is very toxic to aquatic life with long lasting effects. It is a suspected of causing cancer and genetic defects. **Sulfuric acid** causes severe skin burns and serious eye damage. Prolonged and repeated exposure to sulfuric acid mists may cause respiratory irritation and damage to teeth. It is fatal if inhaled. It is harmful to aquatic life. **Acetonitrile** is a highly flammable liquid. It is toxic in contact with skin. **Tetrabutylammonium perchlorate** may cause irritation of the digestive and respiratory tract, skin and eyes. It has a risk of explosion from heat or contamination. It may also react explosively with hydrocarbons and ignite combustibles. **2,2-Dimethoxy-2-phenylacetophenone** may cause irritation of the digestive and respiratory tract, skin and eyes. It is hazardous to the aquatic environment. **Lithium trifluormethylsulfonate** causes skin, eye and respiratory tract irritation. **Poly(ethylenglycol)diacrylate** causes skin corrosion and eye damage. It may cause irritation of the digestive and respiratory tract. Propylene carbonate causes eye and skin irritation.

Electropolymerization as Current Topic in Materials Chemistry

Synthesis of Multifunctional Monomers and Bulk Polymer Networks

All reagents and chemicals were purchased from commercial suppliers, unless otherwise stated. ^{1}H and ^{13}C NMR spectra were obtained on Bruker Avance 400 and III 600 machines. APLI mass spectra were recorded on a Bruker Daltronik micrOTOF system (KrF*-Laser ATLEX-SI, ATL Wermelskirchen). Elemental analyses were obtained on a Perkin Elmer 240 B thermal analyzer. Thermogravimetric analyses were carried out under argon flow on a Mettler Toledo TGA/DSC1 STAR System. UV-vis spectra were recorded on a JASCO V-670. Photoluminescence spectra were recorded on a HORIBA Scientific FluoroMax-4 Spectrofluorometer. Optical measurements were carried out under aerobic conditions at room temperature. A Tousimis Samdri-795 system was used for washing the bulk polymers with supercritical carbon dioxide. Nitrogen and krypton adsorption-desorption isotherms were recorded on a BEL Japan Inc Belsorp-max system at 77 K. A maximum relative pressure of 0.6 was set for measurements with Kr gas. All samples were dried on a Belprep-vac II at 140 °C and ~ 2 Pa overnight prior to the gas sorption measurements.

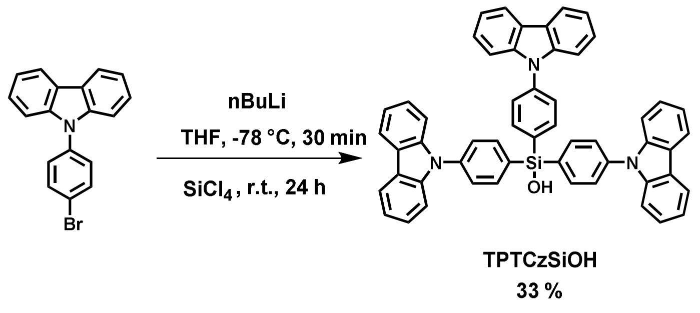

Synthesis of tris(4-(carbazol-9-yl)phenyl)silanol (TPTCzSiOH)

2.48 M n-BuLi in hexane (2.73 mL) was added dropwise to a solution of 9-(4-bromophenyl)-carbazole (2 g, 6.21 mmol) in dry THF (20 ml) at -78°C. After stirring for 1 h, tetrachlorosilane (0.26 g, 1.55 mmol) was added dropwise to the mixture. The mixture was allowed to warm up to r.t. and stirred overnight. After that, a saturated,

aqueous NH_4Cl solution was added for stopping the reaction and the mixture was extracted with DCM. The combined organic phase was washed with water and dried over anhydrous Na_2SO_4. The crude product was purified by column chromatography on silica, (eluent: hexane/chloroform in a gradient from 4:6 to 6:4). Recrystallization from a hexane/chloroform mixture gives a white solid, yield: 0.51 g (33 %). ^1H NMR (600 MHz, $C_2D_2Cl_4$) δ: 8.19 (d, J = 7.7 Hz, 6H), 8.09 (d, J = 8.2 Hz, 6H), 7.78 (d, J = 8.2 Hz, 6H), 7.60 (d, J = 8.2 Hz, 6H), 7.49-7.46 (m, 6H), 7.37-7.34 (m, 6H); ^{13}C NMR (151 MHz, $C_2D_2Cl_4$) δ: 140.30, 139.54, 136.62, 133.43, 126.18, 126.08, 123.28, 120.26, 120.25, 109.87; MS (APLI) 771.270 [771.237] (M^+). Elem. Anal. for $C_{54}H_{37}N_3OSi$, found: C 81.92, H 4.54, N 5.17, rest 8.37; calc: C 84.02, H 4.83, N 5.44, O 2.07, Si 3.64.

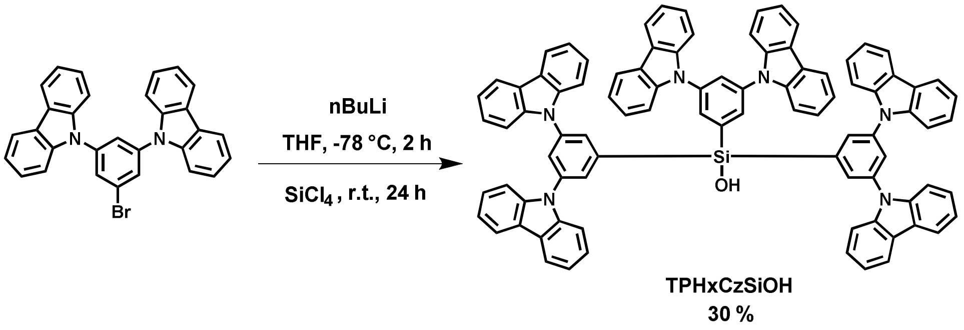

Synthesis of tris(3,5-di(carbazol-9-yl)phenyl)silanol (TPHxCzSiOH)

2.48 M n-BuLi in hexane (1.81 mL) was added dropwise to a solution of 1,3-bis(carbazol-9-yl-5-bromobenzene (2.0 g, 4.10 mmol) in dry THF (20 ml) at -78°C. After stirring for 2 h, tetrachlorosilane (0.174 g, 1.03 mmol) was added dropwise to the mixture. The mixture was then allowed to warm up to r.t. and stirred overnight. After that, a saturated, aqueous NH_4Cl solution was added for stopping the reaction and the mixture was extracted with DCM. The combined organic phase was washed with water and dried over anhydrous Na_2SO_4. The crude product was purified by column chromatography on silica, (eluent: hexane/chloroform in a gradient from 4:6 to 6:4). Precipitation into ethanol from a dichloromethane solution gives a white solid, yield: 0.51 g (30 %). ^1H NMR (400 MHz, $C_2D_2Cl_4$) δ: 8.21 (d, J = 1.96 Hz, 6H), 8.16 (d, J = 7.73 Hz, 12H), 7.98 (t, J = 1.91, 3H), 7.43 (d, J = 8.23, 12H), 7.31-7.26 (m,

12H), 7.18-7.13 (m, 12H); ^{13}C NMR (101 MHz, $C_2D_2Cl_4$) δ: 140.14, 139.56, 137.62, 131.40, 127.48, 126.30, 123.31, 120.56, 120.38, 109.37; MS (APLI) 1267.39 [1267.45] (M+). Elem. Anal. for $C_{90}H_{58}N_6OSi$, found: C 84.26, H 4.40, N 6.51, rest 4.83; calc: C 85.28, H 4.61, N 6.63, O 1.26, Si 2.22.

Synthesis of bulk polymers by oxidative coupling with iron(III)chloride

Both carbazole-based monomers were chemically polymerized in a similar procedure as described now for the TPTCzSiOH monomer: 1,1,2,2-tetra[3',5'-di(carbazol-9-yl)-(1,1'-biphenyl)-4-yl]ethene (0.20 g, 0.201 mmol) was dissolved in anhydrous chloroform (20 mL) and added dropwise to a suspension of iron(III) chloride (0.36 g, 2.20 mmol) in anhydrous chloroform (30 mL) under argon atmosphere. The resulting mixture was stirred at room temperature for one day. After addition of methanol (100 mL), the mixture was stirred for one more hour. The resulting precipitate was collected by filtration and washed with methanol. The powder was treated with aqueous hydrochloric acid (37 %) for 2 h, filtered off and washed with water and methanol. After Soxhlet extraction with methanol and THF for 24 h, the product was treated with ethanol (p.a.) for three days and finally washed with supercritical carbon dioxide. PTPTCzSiOH was isolated as yellow powder, yield: 0.170 g (85 %). PTPHxCzSiOH was also isolated as yellow powder (91 %).

Electrochemical Generation of 3D-Polymer Films and Subsequent Characterization

Acetonitrile and dichloromethane (HPLC grade) were refluxed over calcium hydride and phosphorus pentoxide, respectively, for 3 h and distilled. Tetrabutylammonium perchlorate (TBAP, for electrochemical analysis, ≥ 99.0 %) was purchased from Sigma-Aldrich. Indium tin oxide-coated transparent electrodes on glass (ITO, ≤ 20 Ohm m−2) were purchased from pgo. For electrochemical polymerization and characterization, an electrochemical workstation PAR VersaSTAT 4 was connected to

a QCM922A oscillator (for EQCM experiments) and used in combination to a three-electrode cell. The AFM images were obtained with an atomic force microscope Bruker diInnova operated in tapping mode. The average surface roughness and thickness of the films were extracted from the topography images.

Electrochemical polymerization and characterization on Pt disc and Pt@quartz crystal electrodes

10 mL of 0.1 mM solutions of the monomers were prepared in acetonitrile/dichloromethane (1:4) using 0.1 M TBAP as supporting electrolyte. The solutions were placed in a three-electrode cell under argon atmosphere at 25 °C. A platinum disc electrode (Pt, 1 mm diameter) or platinum spattered quartz crystal electrode (5 mm diameter; nominal frequency 9.00 MHz ± 30 kHz) was used as working electrode (WE); a platinum wire was applied as counter electrode (CE) and Ag°/$AgNO_3$ (0.1 M $AgNO_3$, 0.60 V vs NHE) as reference electrode (RE). A proportionality factor of 1.068 ng Hz^{-1} was determined and used for the analysis of EQCM experiments. Multiple cyclic voltammograms were repeatedly recorded in the potential range of 0 V – 1.1 V with a scan rate of 0.1 Vs^{-1}. The resulting deposits on Pt were placed as WE in 0.1 M solutions of TBAP in acetonitrile. Multiple cyclic voltammograms at different scan rates from 0.005 Vs^{-1} to 0.20 Vs^{-1} were recorded in the potential range of 0 V – 1.1 V.

Electrochemical polymerization on ITO electrodes for porosity measurements and morphological characterization

10 mL of the monomers solutions were placed in a three-electrode cell. ITO (~ 1.5x1.2 cm deposit area) on glass and a platinum gauze (2.5x1.2 cm area), separated by 1 cm, were used as WE and CE, respectively, and Ag°/$AgNO_3$ as RE. For krypton gas sorption measurements, thick films were produced by applying a constant oxidation potential of 1.0 V (TPTCzSiOH), or 1.1 V (TPHxCzSiOH) for 20 min. A potential of -1.0 V was applied after the electropolymerization for 60 s to discharge the deposits.

After rinsing the deposits with acetonitrile and dichloromethane, they were dried for 20 min at 100 °C. The collection of ca. 2 mg of material was necessary for reliable BET measurements. For morphological characterization, MPN films on ITO were prepared by applying ten cyclic voltammograms in the potential range of -1.0 V to 1.0 V (TPTCzSiOH), or -1.0 V to 1.1 V (TPHxCzSiO) at scan rate of 0.01 Vs^{-1}. After rinsing the films with acetonitrile and dichloromethane, they were dried for 20 min at 100 °C.

Electrochemical polymerization on glassy carbon (GC) electrodes for the electrochemical detection of nitrobenzene (NB) as prototypical nitroaromatic analyte

10 mL of a 0.1 mM solution of the monomers were placed in a three-electrode cell under argon atmosphere at 25 °C. A glassy carbon disc electrode (GC, 1 mm diameter) was used as working electrode (WE); a platinum wire as counter electrode (CE); and Ag°/$AgNO_3$ as reference electrode (RE). Potentiostatic polymerization of the monomers was achieved by applying a constant polymerization potential of 1.1 V until an oxidative charge accumulation of 0.20 mC. A potential of -1 V was applied after polymerization during 60 s in order to discharge the deposits. After rinsing the deposits with acetonitrile and dichloromethane, the MPN-modified GC electrodes were used as WE in 10 mL of an aqueous solution of KCl (0.2 M) and PBS (0.1 M) under argon atmosphere at 25 °C. A platinum wire and Ag°/AgCl(sat.) (NaCl 3M, 0.21 V vs NHE) were used as CE and RE, respectively. After 5 min of stirring, a prepotential of 0 V was applied for 30 s. Linear scan voltammograms were recorded from 0 to −1 V with a scan rate of 0.01 Vs^{-1}. For the electrochemical detection of nitrobenzene (NB), aliquots from a 100 M stock solution in acetonitrile were added to the buffered aqueous solutions in order to adjust the NB concentration.

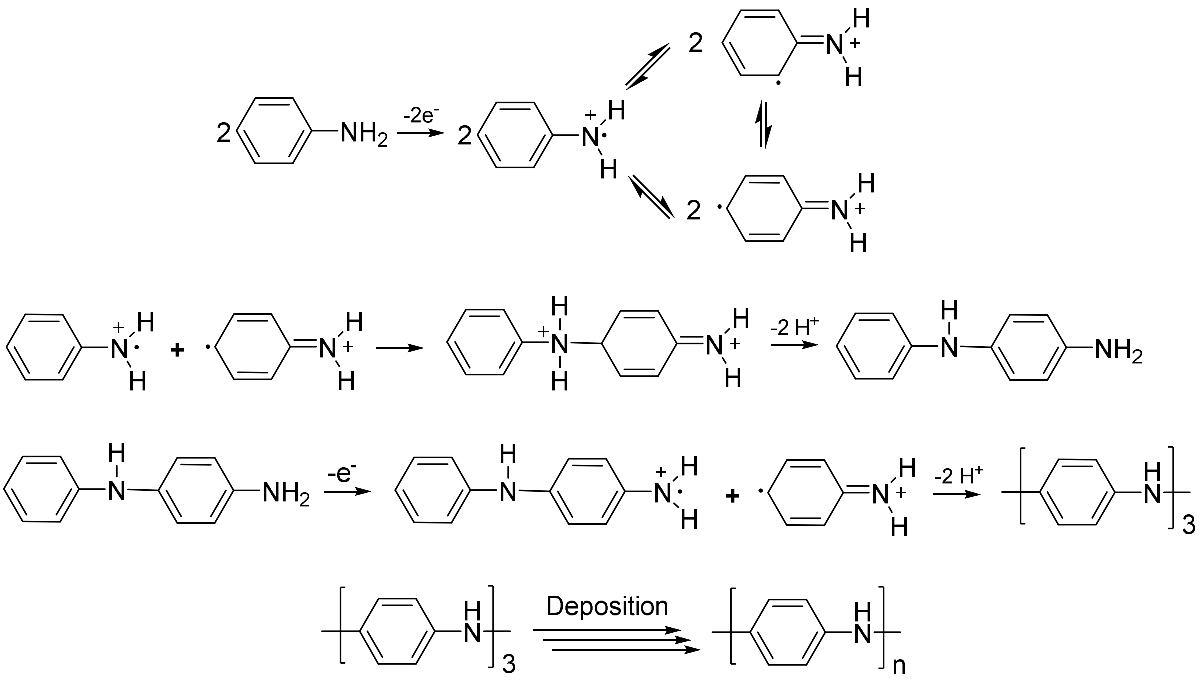

Scheme S1. Electrochemical polymerization steps of aniline condensation during deposition of PANI films [S1]

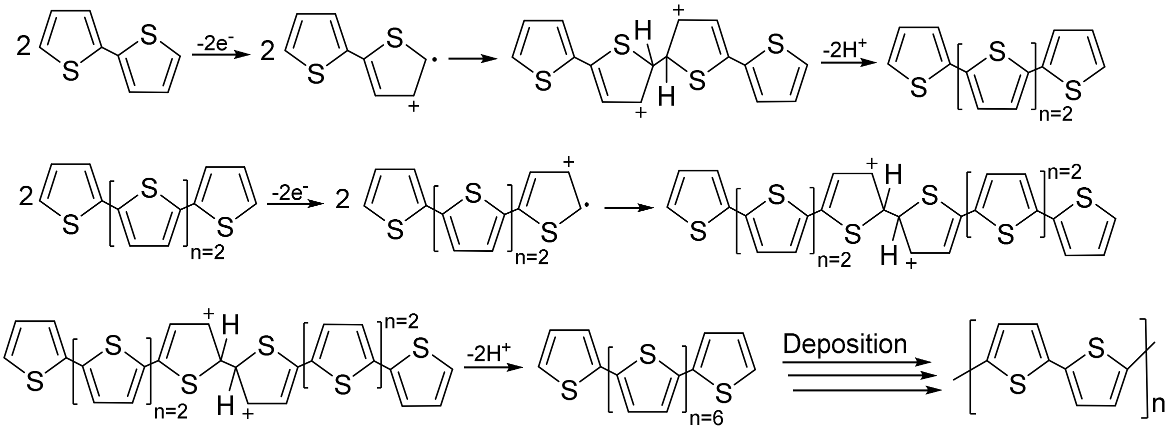

Scheme S2. Electrochemical polymerization steps of bithiophene during deposition of PBTh films [S2]

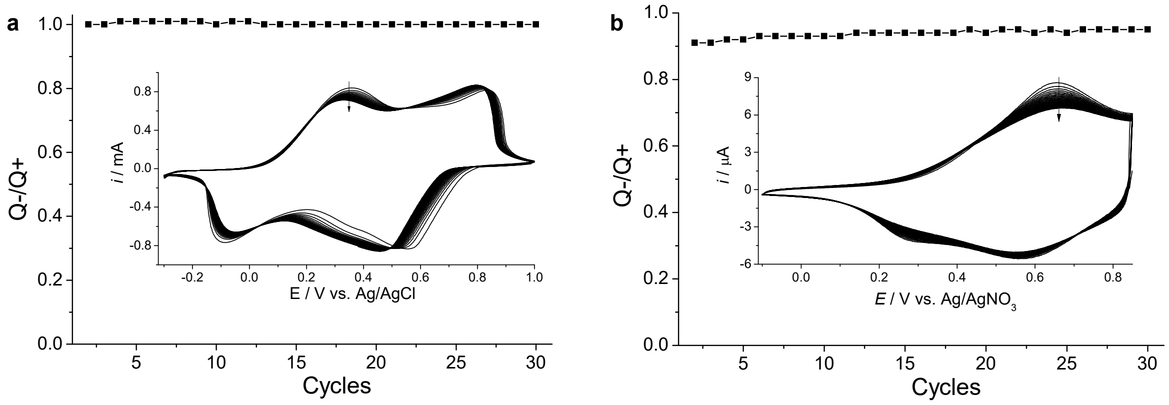

Figure S1. Cathodic to anodic charging/discharging ratios for 30 sweeps (cycles) during deposition of (a) PANI and (b) PBTh onto Pt disc electrodes. Insets show corresponding cyclic voltammograms for both deposited polymer films between -0.1 V and 1.0 V (PANI), or 0.85 V (PBTh) with a scan rate of 0.1 V s^{-1}. The PANI deposition was recorded in a 0.1 M HCl aqueous solution, while PBTh was generated in a 0.1 M tetrabutylammonium perchlorate solution in acetonitrile.

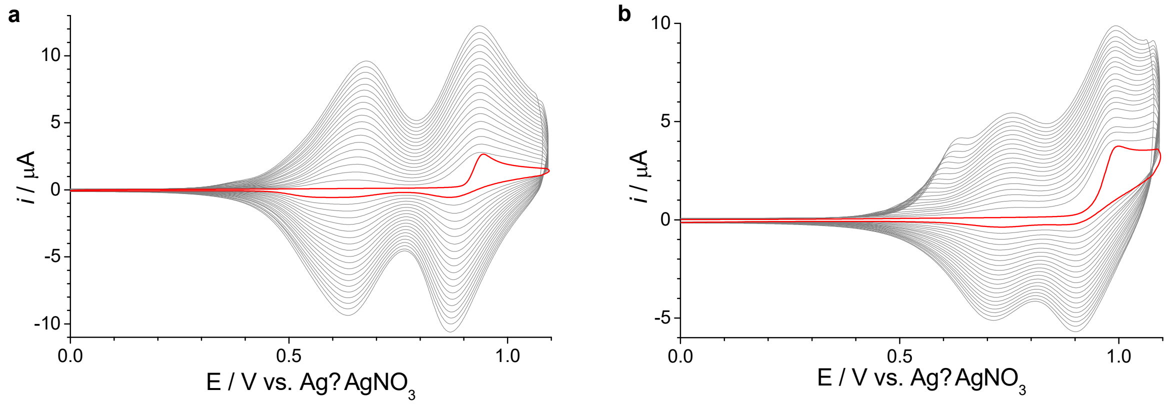

Figure S2. Twenty cyclic voltammograms at Pt disc electrodes carried out for 0.1 mM solutions of (a) TPTCzSiOH, and (b) TPHxCzSiOH monomers in acetonitrile/dichloromethane (1:4) mixtures and 0.1 M TBAP as supporting

electrolyte. Cyclic voltammograms were recorded from -1.0 V to 1.1 V at a scan rate of 0.10 Vs^{-1}.

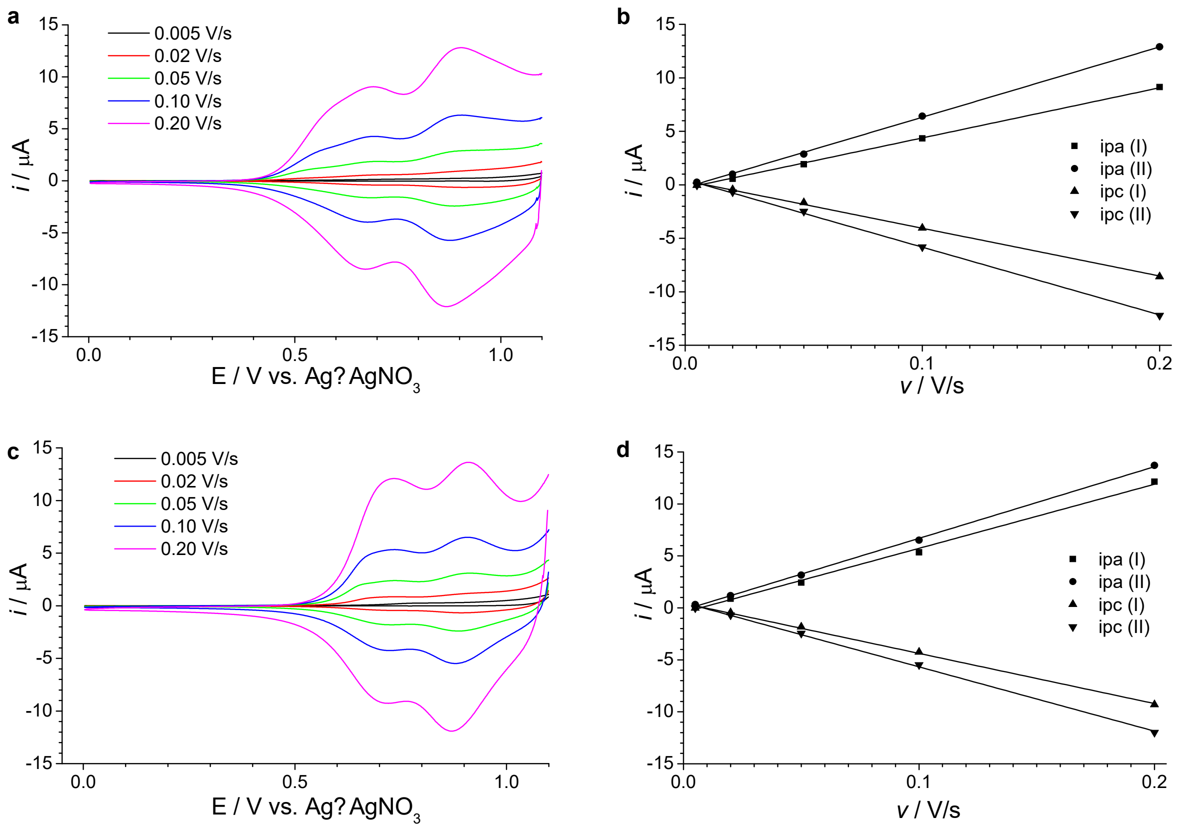

Figure S3. Cyclic voltammograms and linear dependence of peaks current and scan rate for deposits of (a, b) PTPTCzSiOH, and (c, d) PTPHxCzSiOH, respectively, on Pt disc electrodes in monomer-free acetonitrile containing 0.1 M TBAP as supporting electrolyte. A potential range of 0 V – 1.1 V was used at different scan rates from 0.005 to 0.20 Vs^{-1}. The films were prepared as described in the caption of Figure S2.

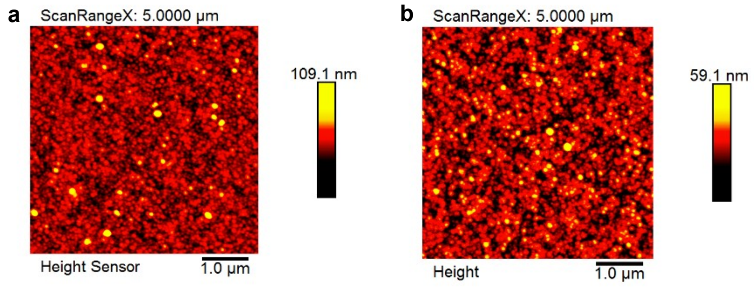

Figure S4. Tapping mode AFM images of MPN films on ITO: (a) PTPTCzSiOH, and (b) PTPHxCzSiOH. The films were electrochemically prepared from a 0.1 mM solution of the monomers in acetonitrile/dichloromethane (1:4) using 0.1 M TBAP as supporting electrolyte. Ten consecutives cyclic voltammograms in the potential range from -1.0 V to (a) 1.0 V or (b) 1.1 V were applied with a scan rate of 0.10 Vs^{-1}.

Table S1. Average roughness (Rq) and thicknesses of electrogenerated MPN films on ITO (for the preparation see the caption of Figure S4).

MPN film	Rq (nm)	Thickness (nm)
PTPTCzSiOH	8.0	45
PTPHxCzSiOH	6.3	50

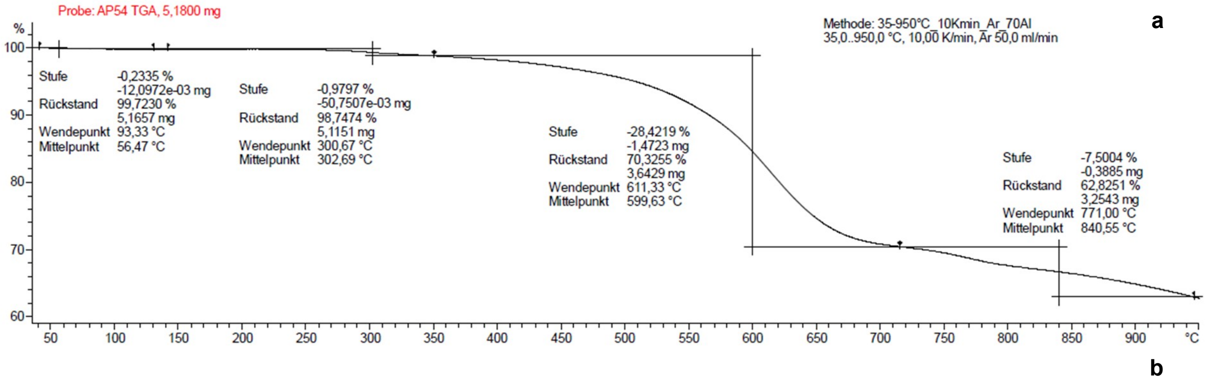

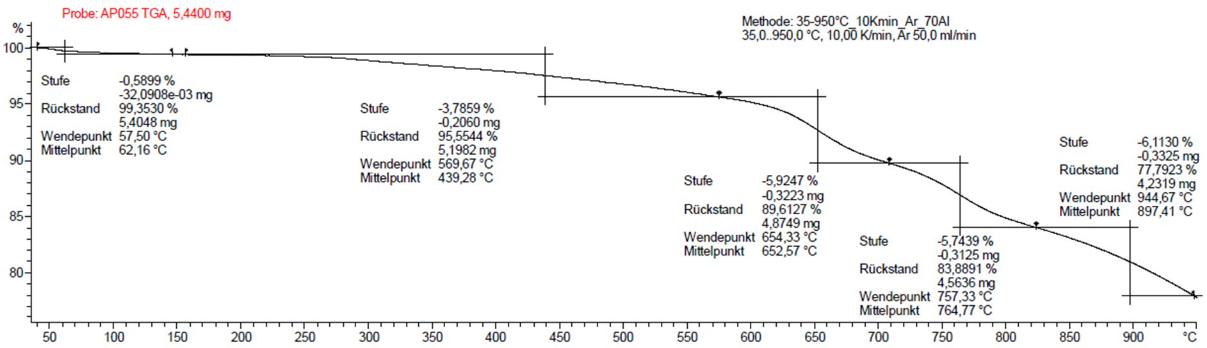

Figure S5. TGA plots of the bulk MPNs (made by $FeCl_3$ oxidation): (a) PTPTCzSiOH, and (b) PTPHxCzSiOH.

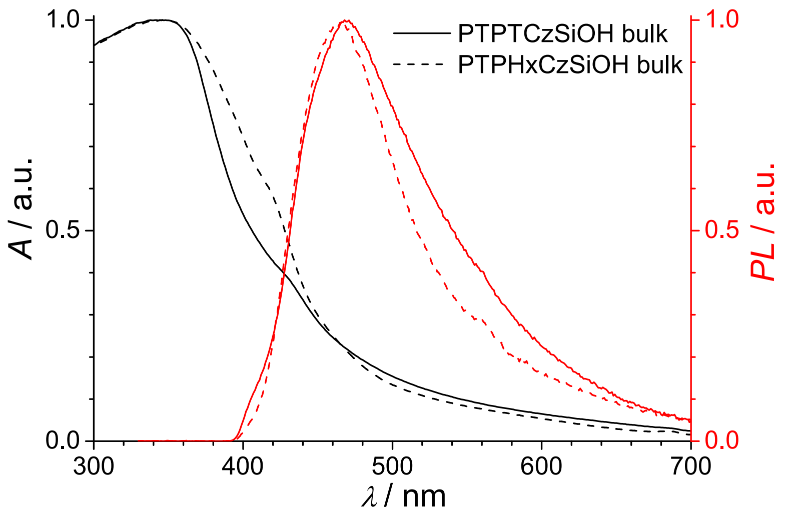

Figure S6. UV-vis (diffuse reflection mode) and PL spectra of the bulk polymers: PTPTCzSiOH (solid line) and PTPHxCzSiOH (dashed line).

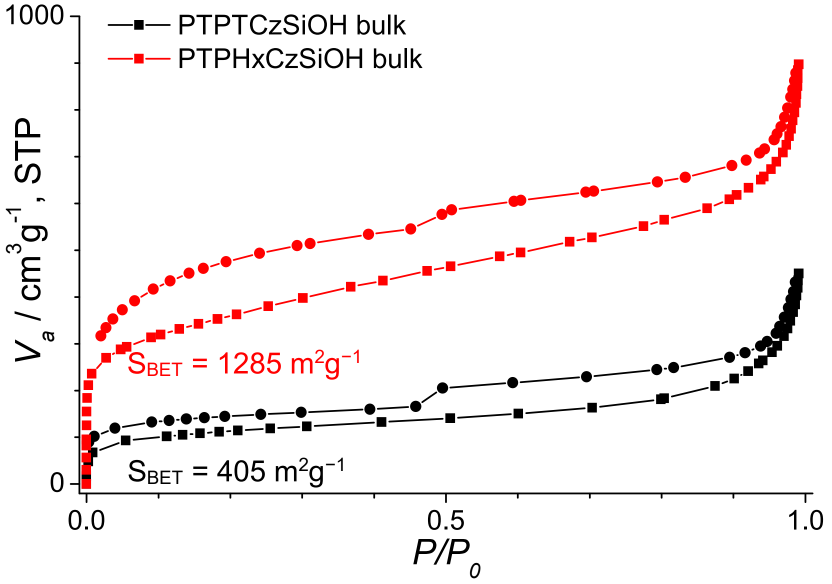

Figure S7. Nitrogen gas sorption isotherms of chemically synthesized bulk MPNs.

References

S1. Molapo, M.K.; Ndangili, M.P.; Ajayi, F.R.; Mbambisa, G.; Milu, M.S.; Njomo, N.; Masikini, M.; Baker, P.; Iwuoha, I.E. Electronics of Conjugated Polymers (I): Polyaniline. *International Journal of Electrochemical Science* **2012**, *7*, 11859-11875.

S2. Heinze, J.; Frontana-Uribe, B.A.; Ludwigs, S. Electrochemistry of Conducting Polymers-Persistent Models and New Concepts. *Chem. Rev.* **2010**, *110*, 4724-4771.
